# Editorial: Endocrine aspects of Noonan syndrome and related syndromes

**DOI:** 10.3389/fendo.2022.1127686

**Published:** 2023-01-05

**Authors:** Giorgio Radetti, Thomas Edouard, Laura Mazzanti, Marco Tartaglia, Martin Zenker

**Affiliations:** ^1^ Marienklinik, Bolzano, Italy; ^2^ Endocrine, Bone Diseases, and Genetics Unit Children’s Hospital, Toulouse University Hospital, Toulouse, France; ^3^ Alma Mater Studiorum, University of Bologna, Bologna, Italy; ^4^ Genetics and Rare Diseases Research Division, Bambino Gesù Children’s Hospital, Rome, Italy; ^5^ Institute of Human Genetics, University Hospital Magdeburg, Magdeburg, Germany

**Keywords:** noonan syndrome (NS), RASopathies, growth hormone, RAS/MAPK pathway, growth, adult height, bone mineral density

Noonan syndrome (NS) is a well-known disorder among pediatric endocrinologists, representing one of the most common non-chromosomal diseases affecting development and growth. NS is characterized by a distinctive gestalt, which is generally associated with congenital and/or evolutive cardiac involvement, variable cognitive deficits, skeletal defects, lymphedema, and increased risk of bleeding and bruising. It belongs to a group of phenotypically overlapping genetic conditions, collectively termed as “RASopathies”, that are caused by pathogenic variants in genes coding for proteins with role in the RAS/mitogen-activated protein kinase (MAPK) signaling pathway. Notwithstanding its substantial genetic heterogeneity and clinical variability, short stature is a major feature of the disorder, representing a common cause for consultation.

Main theme of this special issue of Frontiers in Endocrinology is growth and other endocrinologic aspects in NS and related diseases. Specifically, growth patterns characterizing the RASopathies and the different contributors to growth retardation in these disorders, such as defective/impaired growth hormone (GH) secretion, GH sensitivity, aberrant response to IGF in target tissues, bone metabolism, and genotype as factor influencing the growth rate in response to GH treatment are considered. Furthermore, the efficacy and safety of GH treatment in NS is discussed. As a matter of fact, the authors discuss what is known and what is emerging in NS with the goal of providing information directed to better understand the different aspects of the syndrome and how to improve health care of affected patients.

In their review, Rodriguez et al. evaluate all possible pathophysiological components for the growth delay in patients with NS. They discuss the published evidence regarding spontaneous GH secretion, reduced sensitivity of GH at receptor levels, dysregulated post-receptor signaling, altered autocrine/paracrine IGF-1 production at the growth plate, and finally a direct participation (IGF-1 independent) of the RAS/MAPK pathway on physiological growth plate development. Furthermore, they analyze the factors influencing growth response to GH treatment, as well as the outcomes following rhGH treatment in patients. A critical review of the heterogeneous findings in terms of efficacy of the GH therapy due to the various treatment protocols is provided.

Dahlgren et al. provide an accurate analysis of the 24h GH-profile pattern determined by the PULSAR program and an assessment of GH serum levels in children with NS compared with age-matched cohorts of children with Turner syndrome, and controls (all prepubertal). PULSAR is a program that uses an algorithm to study the episodic hormone secretion and in particular with settings for GH profiles. Response to GH treatment in the NS and Turner syndrome cohorts is also provided. Interestingly, a higher basal and mean GH serum levels, together with lower IGF-1 levels, was documented in the NS cohort compared to a group of control children, suggesting a partial GH insensitivity. NS children also showed disturbances in the secretion pattern of GH with a variable individual response to GH treatment, with higher response to treatment compared to what is observed for Turner syndrome.

Libraro et al. reported the results of a multicenter retrospective study conducted in seven Italian Pediatric Endocrinology centers, evaluating spontaneous growth towards the final height and the effects of rhGH treatment in a large cohort of children with NS, by comparing rhGH-treated versus untreated subjects. Their findings appear encouraging considering both growth response and safety. Notably, they note a marked loss in height SDS within the first two years of life, pointing out the importance of an early start of rhGH treatment. In NS with GH deficiency (GHD), rhGH treatment at the standard dose used for children with GHD seems effective in improving growth and adult height. The authors, however, suggest the need of further studies to assess genotype-specific response to rhGH treatment.

Stagi et al. provide an updated overview of the growth pattern and contributing factors (e.g., GH-deficiency, partial GH insensitivity, and altered response to IGF-1) of growth failure in patients with NS. They also provide a systematic analysis of the available data collected in terms of response to rhGH treatment and risk for evolutive complications. While the collected data document a consistent and significant increase in height velocity (HV) in NS children and adolescents treated with rhGH, the authors point out the importance of a personalized treatment with rhGH taking into account the genotype and an individualized follow-up and close monitoring during treatment.

Siano et al. evaluated the prevalence of endocrine disorders in a cohort of patients with a genetically confirmed RASopathy including in the study an age- and sex-matched healthy control group. Short stature was detected in half of the patients with NS and CFCS. The high prevalence of thyroid autoimmunity confirmed that these patients had an increased risk to develop autoimmune disorders. However, the presence of thyroid autoantibodies was associated to normal thyroid function, and it was hypothesized that these antibodies can precede the clinical symptoms of the disease and could be used for diagnostic and prognostic purposes. Both NS and CFCS patients showed lower BMD than controls. Reduced BMD was associated to reduced physical activity, joint pain and high levels of inflammatory cytokines. These findings are expected to have implications for the follow-up and prevention of osteopenia/osteoporosis in both NS and CFCS.

In summary, this Research Topic represents a snapshot on the multiple facets of current knowledge on endocrine aspects of NS. It points out the recent achievements in endocrinological health care of these patients, but also unmet needs on the way towards personalized medicine.

## Author contributions

All authors listed have made a substantial, direct, and intellectual contribution to the work and approved it for publication.

